# Health-related quality of life and extent of self-care practice among heart failure patients in Ethiopia

**DOI:** 10.1186/s12955-020-01290-7

**Published:** 2020-02-14

**Authors:** Mohammed Assen Seid

**Affiliations:** Department of Clinical Pharmacy, University of Gondar, Gondar, Ethiopia

**Keywords:** Quality of life, Self-care, Heart failure, Ethiopia

## Abstract

**Background:**

Heart Failure (HF) results in an immense impact on the patients’ Health-related quality of life (HRQOL). Heart failure patients HRQOL is dependent on the patients’ level of engagement in self-care behaviors. Therefore this study aimed to determine HF patients’ health-related quality of life and its relationship with self-care behaviors.

**Methods:**

An institutional-based study was conducted on 284 heart failure patients at the University of Gondar referral hospital. The data were collected using a structured questionnaire-based interview. The data were analyzed using SPSS version 20. Both descriptive and analytical statistical tests were utilized. A multinomial logistic regression analysis was done to determine the association between HRQOL and different independent variables. Variables with a *p*-value< 0.05 were considered as a significant predictor of the outcome variable.

**Results:**

The finding of this study showed that more than sixty-six percent of the study population were females. The overall mean score of HF patients’ quality of life was 46.4 ± 22.4 and the physical and emotional subscale mean score was 20.2 ± 9.8 and 10.5 ± 6.8 respectively. The majority of the study participants 147(51.8%) had poor quality of life. The multinomial logistic regression analysis result showed that rural residence (odds ratio 2.41, 95% CI, 1.23 to 4.71) and inadequate level of self-care practice (odds ratio 2.61, 95% CI, 1.43 to 4.78) were independent predictors of poor HRQOL. The correlation analysis also showed that there was a significant negative relationship between HF patients’ HRQOL score and Self-care practice score (r = − 0.127, *P* = 0.032).

**Conclusion:**

Overall, the majority of HF patients had poor HRQOL. Heart failure patients’ HRQOL was significantly associated with place of residence and patients’ level of self-care practice. Therefore, patients with HF are required to learn the benefit of self-care behaviors to improve their quality of life and to decrease the disease progression. Furthermore, HF patients who come from rural areas need special emphasis in each follow-up evaluation.

## Introduction

Heart failure is a progressive clinical syndrome affecting more than 38 million people globally (1, 2). HF leads to significant morbidity, mortality and has an immense impact on the patients’ health-related quality of life (HRQOL) (3–6). Health-related quality of life (HRQL) is a general concept that represents the patient’s overall perception of the impact of an illness and its treatment. At a minimum, it reflects physical, psychological (including emotional and cognitive), and social functioning (7). Health-related quality of life (HRQOL) is substantially more impaired in patients with HF than patients with other chronic diseases (8, 9).

The principle of HF management focuses on prolonging HF patients’ life by maintaining physiological stability (10) and improving HRQOL (11). Despite the advancement of heart failure management, HF related hospitalization and mortality are increasing (12). It is assumed that HF patients’ involvement in the daily self-care behavior activities, including adherence to medication, monitoring of signs and symptoms and lifestyle modifications had a great influence on the disease progression (13, 14). These self-care behavior activities among heart failure patients vary considerably in Africa, including Ethiopia (15–17).

Heart failure patients’ HRQOL is dependent on the patients’ level of engagement in self-care behaviors (18). Self-care behavior is a modifiable factor that emphasizes the actions to be taken by HF patients to maintain life, healthy functioning and to improve the overall HRQOL (19–21). Appropriate self-care behavior in HF patients is assumed to have a good outcome in the overall disease progression. However, based on previous studies, the evidence is inconsistent for health-related quality of life (HRQOL) (22–25).

Improving HRQOL is one of the primary objectives in the management of patients with heart failure (26). Therefore, having a comprehensive understanding regarding the relationship between HF patients’ self-care behaviors and their level of HRQOL will support to develop tailored interventions to reduce symptom burden and improve patient quality of life (18).

To the best of the author’s knowledge, there was no evidence regarding HRQOL in heart failure patients in Ethiopia. Hence, the aim of this study was to determine HF patients’ health-related quality of life and the relationship with self-care behaviors.

## Methods

### Study setting and period

An institutional-based prospective cross-sectional study was conducted at the University of Gondar referral hospital from March to June 2017. The University of Gondar is located in Gondar town, Northwest Ethiopia. Heart failure patients had once per week follow up services in the outpatient department (OPD) of the hospital. In this study, patients who were 18 years old or above, had been diagnosed with HF, and start to take medications and had at least 1 month of follow up were included. Data were collected from a total of 284 heart failure patients who were included in this study.

### Data collection tools and procedures

Structured and validated tools that were adopted from previous studies were used for data collection (19, 27, 28). The prepared questionnaire had four different sections, which include socio-demographic characteristics, clinical characteristics, Quality of life, and Self-care components. Heart failure-specific health-related quality of life (HRQOL) was assessed by using the Minnesota Living with HF Questionnaire (MLHFQ), a 21-item scale that has a physical (8 items) and an emotional (5 items) subscales. This MLHFQ used to evaluate how much the disease and its treatment had affected the patient’s life in the last month (4 weeks). The MLHFQ is a valid and reliable tool extensively used to assess HRQOL in HF patients. The 21-items have a 6-point Likert scale ranging from 0 (no effect) to 5 (very much). The maximum total score of the MLHFQ is 105, with a higher score indicating worse HRQOL. HF patients who score less than 24 labeled as having (Good) HRQOL, 24–45 (Moderate), and greater than 45 as (Poor) HRQOL (29–32).

The European Heart Failure Self-care Behavior scale (EHFScBS-9) questionnaire was used for the assessment of self-care behavior. All the EHFScBS-9 items had a 5-point Likert scale from 1 (“completely agree”) to 5 (“completely disagree”). This scale has two components the adherence (weight monitoring, limit the amount of fluid intake, follow a low-sodium diet, take their medications as prescribed, and regular exercise) and the consulting behaviors (HF patients contact their doctor/nurse in case of shortness of breath, legs/feet swelling, weight gain, and fatigue). The possible score of this scale varies from 9 to 45, with a lower score indicating better self-care. To make the interpretation of the EHFScBS-9 easier, each Likert scale is reversed to 1 (“completely disagree”) to 5 (“completely agree”) then converted to 0 to 100 standardized score based on this formula ((total score-9)*2.7777) from the previous study and with a higher score indicating better self-care (28). Furthermore in this study self-care behavior of HF patients was classified as adequate (above the mean score) and inadequate (below the mean score). Data were collected through a structured interview by trained pharmacists and nurses.

### Data analysis and interpretation

All the collected data were checked for completeness and consistency of responses manually. After cleaning, data were coded, entered into Epi Data version 3.1 and finally analyzed using SPSS version 20. Both descriptive and analytical statistical tests were utilized. The multinomial logistic regression analysis was done to determine the association between HRQOL and different independent variables. Independent variables with a *p*-value< 0.05 were considered as a significant predictor of the outcome variable.

## Results

### Socio-demographic characteristics

More than half of the study participants (159,56%) were above the age of 50 years. The majority were females (187,65.8%) and more than half were married (149,52.5%). Of all participants, (175,61.6%) of HF patients had not attended formal education, and (130, 46%) were living in rural areas (Table 1).

**Table 1:** Socio-demographic characteristics of heart failure patients at the University of Gondar referral hospital

Variables	Frequency	Percentage
Age (in years)		
< 50	125	44
≥ 50	159	56
Sex		
Male	97	34.2
Female	187	65.8
Marital status		
Married	149	52.5
Single	55	19.4
Divorce	31	10.9
Widowed	49	17.3
Educational level		
No formal Education	175	61.6
1–8	57	20.1
9–12	33	11.6
College/University	19	6.7
Occupation		
Farmer	58	20.4
Housewife	109	38.4
Merchant	12	4.2
Student	28	9.9
Retired	10	3.6
Daily labor	67	23.6
Place of residence		
Urban	154	54.2
Rural	130	45.8

### Clinical characteristics of the study participants

In this study, nearly half (134,47.2%) of the study participants had chronic disease comorbidity. The majority (169,59.5%) had less than 5 years of history with the diseases and around two thirds (179,63%) of the study participants had a history of hospitalization due to HF (Table 2).

**Table 2:** Clinical characteristics of the study participants at the University of Gondar referral hospital

Variable	Frequency	Percentage
Chronic comorbidity		
None	150	52.8
Yes	134	47.2
Duration since diagnosed for heart failure (in years)		
< 5 years	169	59.5
5–10	86	30.3
> 10 years	29	10.2
Duration since started to take HF medications		
< 5 years	191	67.3
5–10	71	25.0
> 10 years	22	7.7
Hospitalization history		
No	105	37.0
Yes	179	63.0
Total number of prescribed medication per day (Mean ± SD)		3.11 ± 1.104.

### Health-related quality of life (HRQOL) of heart failure patients

In this study, The mean score of HF patients’ quality of life was 46.4 ± 22.4. The mean physical and emotional subscale scores were 20.2 ± 9.8 and 10.5 ± 6.8, respectively. The majority (147,51.8%) of the study participants had poor Quality of life (Table 3). The results of the multinomial logistic regression analysis presented in (Table 4) showed that place of residence (odds ratio 2.41, 95% CI, 1.23 to 4.71) and levels of self-care practice (odds ratio 2.61, 95% CI, 1.43 to 4.78,) were independent predictors of poor HRQOL.

**Table 3:** level of heart failure patients’ quality of life at the University of Gondar referral hospital

Heart failure patients HRQOL score	Median (IQR)	Mean (±SD)
Total MLHFQ score (range 0–105)	47 (42–52)	46.4 ± 22.4
Physical score (range 0–40)	21 (18–23)	20.2 ± 9.8
Emotional score (range 0–25)	10 (9–12)	10.5 ± 6.8
Levels of quality of life		
Good	58	20.4%
Moderate	79	27.8%
Poor	147	51.8%

*MLHFQ* Minnesota Living with HF Questionnaire

**Table 4:** Multinomial logistic regression analysis for factors associated with heart failure patients’ quality of life (with moderate HRQOL as the reference category)

Variables	Level of HRQOL							
	Good				Poor			
	Odds ratio	95% CI		p-value	Odds ratio	95% CI		*p*-value
Age (in years)								
< 50	1.11	0.43	2.88	0.826	1.32	0.58	2.99	0.507
≥ 50	_				_			
Sex								
Female	0.93	0.39	2.16	0.857	1.32	0.66	2.61	0.432
Male	_				_			
Marital status								
Single	0.24	0.04	1.31	0.100	1.00	0.26	3.79	0.999
Married	1.28	0.45	3.59	0.644	1.18	0.49	2.79	0.715
Divorce	2.45	0.51	11.77	0.264	2.72	0.68	10.80	0.156
Widowed	_				_			
Educational level								
No formal Education	0.41	0.09	1.86	0.247	0.62	0.16	2.42	0.492
1–8	0.49	0.11	2.31	0.368	0.56	0.15	2.18	0.406
9–12	0.96	0.18	5.21	0.963	1.09	0.26	4.65	0.906
College/University	_				_			
Place of residence								
Rural	0.89	0.392	2.07	0.802	2.41	1.23	4.71	0.010*
Urban	_				_			
Chronic comorbidity								
None	1.33	0.60	2.93	0.479	0.54	0.28	1.04	0.067
Yes	_				_			
Hospitalization history								
No	1.55	0.75	3.23	0.238	1.12	0.60	2.08	0.727
Yes	_				_			
Self-care practice								
Inadequate	0.99	0.47	2.08	0.969	2.61	1.43	4.78	0.002*
Adequate	_				_			

*Statistically significant (*p* < 0.05)

### Heart failure patients’ self-care practices

This study showed that around 48% of the participants had inadequate self-care practices. It also revealed that heart failure patients give more emphasis on selected self-care recommendations. Such as, if they experience shortness of breath (mean score 4.75), and a sign of legs/feet edema (4.49) they would contact their doctor or nurse more frequently and the majority of them also took their medication as prescribed. In contrast, HF patients had poor self-care practices towards regular exercise (mean score 2.21), limit the amount of fluid intake (1.32), and weight monitoring (1.55) (Table 5).

**Table 5:** Mean EHFScBS-9 items self-care practice score in rank order in patients with HF at the University of Gondar referral hospital

EHFScBS-9* items	Mean score of each self-care
1. If SOB increases I contact my doctor or nurse	4.75
2. I take my medication as prescribed	4.74
3. If legs/feet are more swollen, I contact my doctor or nurse	4.49
4. I eat a low-salt diet	3.86
5. If I experience fatigue I contact my doctor or nurse	3.56
6. If I gain weight more than 2 kg in 7 days I contact my doctor or nurse	2.89
7. I exercise regularly	2.21
8. I weigh myself every day	1.55
9. I limit the amount of fluids	1.32
Overall self-care practices	
Adequate	148 (52.1)
Inadequate	136 (47.9)

EHFScBS-9- The European Heart Failure Self-care Behavior scale- a nine-item scale, SOB-shortness of breath.

### The relationship between HF patients’ HRQOL and self-care practice

**Fig. 1:**
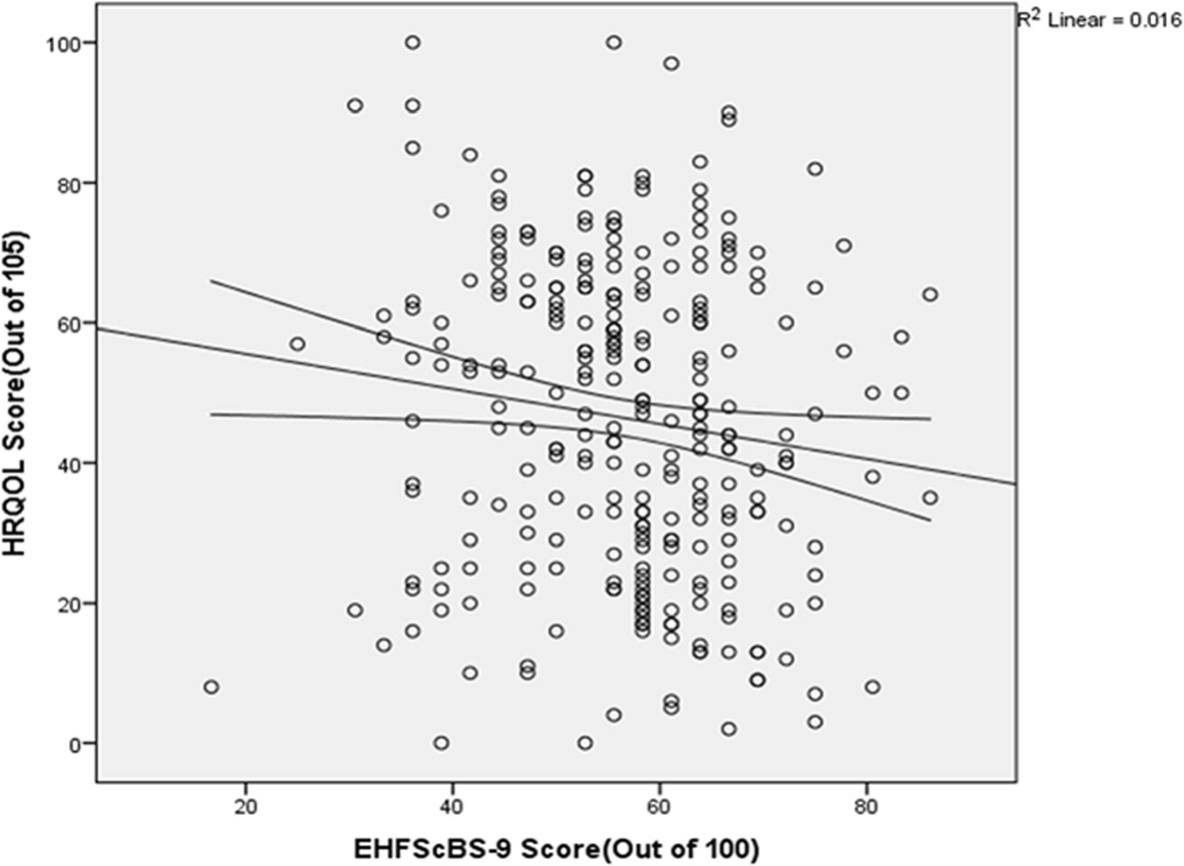
The correlation between HRQOL score and Self-care practice score among heart failure patients at the University of Gondar Referral Hospital

The result of this study showed that there was a significant negative relationship between HF patients’ HRQOL score and Self-care practice score (r = −0.127, P = 0.032). When HF patients’ self-care practice score increases (good self-care) their HRQOL score decreases (Good Quality of life) (Fig. 1).

## Discussion

The ultimate goal in HF management is not merely focused on the patients’ survival but also on the improvement of their HRQOL (31, 33). This study aimed to assess HF patients’ HRQOL and its association with the patients’ level of engagement towards self-care recommendations. Quality of life in patients with HF was significantly impaired in all dimensions (physical functioning and emotional subcomponent). The finding of this study revealed that the majority of heart failure patients (52%) had poor HRQOL (95% CI, 46.2–58.1%). This quality of life score implicates that the majority of heart failure patients’ life in this setup is much more affected by the disease condition and its management.

This study also showed that nearly half (48%) of HF patients’ self-care engagement was found to be inadequate. The mean score (Table 5) for each self-care recommendations showed that HF patients contact their health care provider more frequently when they experience shortness of breath (SOB) than other symptoms. The majority of HF patients also take their medications as prescribed. However, the finding of this study implicates that HF patients had poor self-care engagement towards performing regular exercise, monitoring their weight and limiting the amount of fluid intake. Therefore healthcare providers should give more emphasis on these self-care recommendations during each follow-up evaluation.

In this study, the patients’ place of residence and their level of self-care practice had a statistically significant association with HRQOL. HF patients who came from rural areas had 2.4 times poorer quality of life than those living in urban areas (OR = 2.41, 95% CI, 1.23–4.71). This might be due to the fact that patients in rural areas had low literacy levels to practice each self-care recommendation and the presence of different challenges in this area impede them to get high-quality healthcare service which leads to poorer HRQOL than urban HF patients (23).

Regarding the association between self-care and HRQOL, previous studies reported inconclusive results (23–25, 34, 35). The result of the current study supports the existence of a significant association between self-care behaviors and HRQOL. It is understood that HF patients who had inadequate self-care practices had 2.6 times poorer HRQOL than patients who had adequate self-care engagement (OR = 2.61, 95%CI, 1.43–4.78). The correlation analysis also showed that there is a significant negative relationship between HF patients’ HRQOL and Self-care practice scores (r = − 0.127, *P* = 0.032). When HF patients had poor self-care practice their quality of life become worse. This finding is consistent with other similar studies report, heart failure patients who had poor self-care engagement had worse HRQOL (18, 24, 30, 34, 36, 37). Kessing et al. (30) also reported that lower self-care was associated not only with the overall HRQOL but also with physical and emotional subcomponents of quality of life. The result of the present study provides an insight into the inconclusive association among self-care behaviors and HRQOL. Further prospective, follow-up based research is recommended in the future to elucidate the temporal relationships and other factors associated with HRQOL. In addition to this, considering qualitative research will be one of the best methods to generate data which support the comprehensive understanding of their self-care behaviors and their quality of life from the perspective of each study participants, and to uncover their beliefs, values, and motivations that underlie the individual health behaviors (38).

Even though this study was the first study to assess HF patients’ HRQOL and its association with self-care behavior in Ethiopia, it was not out of limitations. It is a single-center study, the presence of social desirability and the recall bias during self-report might have affected the data obtained. The cross-sectional nature of the study design cannot delineate a causal relationship between quality of life and self-care. Therefore, it is better to take into consideration these limitations while interpreting the finding of this study.

## Conclusions

The majority of HF patients attending this hospital had poor HRQOL and nearly half of the patients also had inadequate levels of self-care practices. Mainly, HF patients had poor self-care behavior towards performing regular exercise, weight monitoring and limitation of fluid intake. Worse HRQOL had a statistically significant association with inadequate self-care practice and rural residence. It implicates that health care providers should work on the improvement of HF patients’ self-care engagement to improve their quality of life and to decrease the overall disease progression.

## Data Availability

All data generated or analyzed during this study are included in this published article.

## Abbreviations

C. I: Confidence Interval
EHFScBS-9: European Heart Failure Self-care Behavior Scale
HF: Heart Failure
HRQOL: Health-Related Quality Of Life
IQR: Interquartile Range
MLHFQ: Minnesota Living with HF Questionnaire
OR: Odds Ratio
SD: Standard Deviation
SOB: Shortness of breath
SPSS: Statistical Package for the Social Sciences
